# Extracellular signal-regulated kinase 1/2 is required for complement component C1q and fibronectin dependent enhancement of Fcγ- receptor mediated phagocytosis in mouse and human cells

**DOI:** 10.1186/s12865-020-00393-6

**Published:** 2020-12-14

**Authors:** Emily A. Willmann, Vesna Pandurovic, Anna Jokinen, Danielle Beckley, Suzanne S. Bohlson

**Affiliations:** 1 Department of Microbiology and Immunology, Des Moines University, Des Moines, IA USA; 2 Department of Molecular Biology and Biochemistry, University of California, Irvine, CA USA

**Keywords:** C1q, Complement, Fibronectin, Phagocytosis, ERK1/2, Fcγ-receptor, TNFα

## Abstract

**Background:**

C1q is a soluble pattern recognition protein that regulates multiple leukocyte functions, and deficiency in C1q results in autoimmunity. C1q stimulates enhanced phagocytic function through multiple mechanisms including the rapid enhancement of Fcγ receptor (FcγR) -mediated phagocytosis. The molecular mechanism responsible for this rapid enhancement of phagocytic function is unknown. The purpose of this study was to investigate the molecular pathway required for C1q-dependent enhanced phagocytosis.

**Results:**

Leukocyte associated immunoglobulin like receptor-1 (LAIR-1) is a receptor that mediates C1q-dependent activation of leukocytes; however, using LAIR-1 deficient mouse bone marrow derived macrophages (BMDM), we demonstrated that LAIR-1 was not required for C1q-dependent enhanced FcγR-mediated phagocytosis. A phospho-kinase array identified extracellular signal-regulated kinase (ERK) 1/2 as dysregulated following activation with C1q. Validation of the array in BMDM and the human monocyte cell line THP-1 demonstrated a decrease in basal ERK1/2 phosphorylation in C1q-stimulated cells compared to control cells. However, subsequent stimulation with immune complexes stimulated rapid upregulation of phosphorylation. The extracellular matrix protein fibronectin regulates enhanced phagocytic activity in macrophages similar to C1q, and both C1q and fibronectin-dependent enhanced phagocytosis required ERK1/2 since both were blocked by pharmacologic inhibition of ERK1/2. Furthermore, diminished C1q-dependent ERK1/2 phosphorylation was sustained after four-hour treatment with lipopolysaccharide and correlated with a significant reduction in TNFα production.

**Conclusions:**

These data demonstrate that C1q and fibronectin utilize a similar ERK1/2-dependent mechanism for enhanced phagocytosis, which should lead to development of novel approaches to modulate C1q-dependent regulation of macrophage activation, inflammation and autoimmunity.

**Supplementary Information:**

The online version contains supplementary material available at 10.1186/s12865-020-00393-6.

## Background

C1q, the recognition component of complement component C1, functions independently of the complement cascade as a soluble pattern recognition protein. C1q is a 460 kDa glycoprotein consisting 18 polypeptide chains that assemble to form a globular heads region and collagen like tail. C1q binds to a wide range of structures and elicits responses from myeloid cells, such as the modulation of proinflammatory cytokine production and enhancement of phagocytic function [reviewed in [1]]. C1q enhances phagocytic function through a variety of mechanisms including activation of the classical complement pathway and subsequent opsonization by complement cleavage products such as C3b and C4b, direct opsonization by C1q, and upregulation of engulfment machinery through cellular programming of macrophages [reviewed in [2]]. C1q-dependent enhancement of engulfment of apoptotic cells has been the focus of significant research and is likely important in prevention of autoimmunity associated with C1q deficiency [1]. In addition to its role in engulfment of apoptotic cells, C1q enhances engulfment of targets suboptimally opsonized with antibody or complement, and this may be important in promoting host defense during primary immune responses and/or in immunocompromised individuals [3, 4]. The collagen-like tail of C1q is sufficient for triggering C1q-dependent phagocytosis of suboptimally opsonized targets [4], and molecules with similar structure such as mannose binding lectin (MBL), also trigger enhanced phagocytosis [5]. A consensus motif required for enhanced phagocytosis (GE(K/Q/R)GEP) was identified within the collagen-like tail using recombinant MBL and mutants thereof [6]. The globular head regions of C1q bind to target particles, such as immunoglobulins [7] and apoptotic cells [8] enabling C1q to bridge the target and the phagocyte.

While numerous potential C1q receptors have been described, the receptor(s) that mediates C1q-dependent enhancement of phagocytosis has not been definitively identified. LAIR-1, a collagen receptor on leukocytes, has been reported to mediate some C1q-dependent functions, such as the C1q-dependent inhibition of proinflammatory cytokine production in human monocytes and dendritic cells, as well as C1q-dependent differentiation of monocytes to an M2-like phenotype [9–11]. The LAIR-1 cytoplasmic tail contains an immunoreceptor tyrosine based inhibitory motif (ITIM) that when phosphorylated, recruits the tyrosine-specific protein phosphatase, SHP-1. Activation of SHP-1 negatively regulates intracellular signaling and is important in protection from chronic inflammation [12]. Furthermore, C1q mediates the crosslinking of LAIR-1 with CD33, an inhibitory immunoreceptor on monocytes responsible for cell activation and differentiation [13]. Recently, ligation of LAIR-1 with either collagen or C1q was demonstrated to inhibit LAIR-1 phosphorylation and subsequent T-cell signaling following T-cell receptor activation [14]. The role of LAIR-1 in regulation of C1q-dependent phagocytic function has not been previously explored.

C1q-dependent enhancement of Fcγ receptor (FcγR)-mediated phagocytosis occurs within 5 min of stimulation with C1q [15]. This enhanced phagocytic function does not require de novo protein synthesis since it is not inhibited by cycloheximide [16]. Although numerous studies have demonstrated C1q-dependent enhancement of FcγR-mediated phagocytosis in phagocytes from multiple species and differentiation states, including human, mouse and rat cells [4, 17, 18], the impact of C1q on signaling through the FcɣRs has not been fully explored. Extracellular signal-regulated kinase 1 and 2 (ERK1/2) are related protein-serine/threonine kinases of the mitogen-activated protein kinases (MAPK) family that participate in intracellular signaling cascades responsible for regulation of a large number of cellular processes, including innate immune responses induced by signaling through the FcγRs such as phagocytosis, generation of reactive oxygen species and cytokine production (reviewed in [19]). However, the downstream effects of ERK1/2 activation are conditionally determined. For example, mouse bone-marrow derived macrophage (BMDM) activation with immune complexes (IC) is dose dependent. High density immune complex stimulation leads to enhanced ERK1/2 activation and production of anti-inflammatory IL-10 and a reduction in pro-inflammatory IL-12, whereas stimulation with lower doses of IC results in the opposite reaction (high IL-12 and low IL-10) [20]. Furthermore, inhibition of ERK1/2 reduced phagocytosis of apoptotic cells in J774 macrophages but did not reduce FcγR-mediated phagocytosis [21].

Here, we utilized a phospho-kinase array and subsequent validation with western blotting to identify C1q-dependent regulation of ERK1/2 phosphorylation. Inhibitor analysis demonstrated that ERK1/2 is an essential intermediate in C1q-dependent FcγR-mediated phagocytosis in both mouse and human phagocytes. Moreover, we demonstrated that this signaling pathway was shared with the extracellular matrix protein fibronectin, a protein previously demonstrated to enhance phagocytic activity similar to C1q [22]. Combined, these studies should foster continued characterization of C1q dependent signaling pathways in phagocytes.

## Results

### C1q stimulates enhanced FcγR-mediated phagocytosis in multiple macrophage populations

**Fig. 1:**
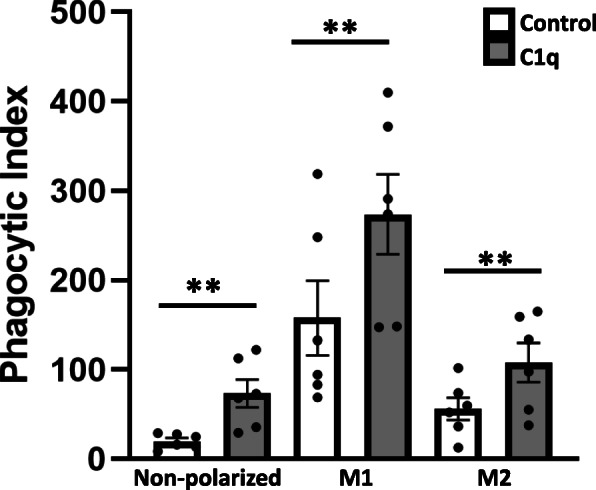
M1 and M2 polarized BMDM respond to C1q with an enhancement of phagocytosis. BMDM were either nonpolarized or polarized overnight to an M1 phenotype (classically activated) or an M2 phenotype (alternatively activated) and then adhered to 4 μg/mL HSA (control, white bars) or C1q (grey bars) for 30 min. BMDM were then incubated with EAIgG for an additional 30 min. Scoring was performed blinded with at least 200 cells per well scored. The phagocytic index was calculated as the total number of red blood cells in 100 macrophages, multiplied by 100. Bars represent the average of two individual wells from three separate experiments ± standard error of the mean. Each point represents a replicate. ** *p* ≤ 0.01, paired student’s t-test

C1q-dependent enhancement of FcγR-mediated phagocytosis has been demonstrated in multiple species including human [4], rat [18] and mouse cells [17]. To determine if macrophage polarization altered responsiveness to C1q, mouse BMDM were polarized toward an M1 phenotype using IFNγ and LPS, an M2 phenotype using IL-4, or left non-polarized. Polarized and non-polarized BMDM were adhered to a control protein (HSA) or C1q for 30 min and then cultured with sheep red blood cells suboptimally opsonized with IgG (EAIgG) for an additional 30 min. Non-polarized BMDM which have previously been shown to respond to C1q with an enhancement of phagocytosis were used as a positive control [17]. As expected, M1 macrophages ingested more EAIgG irrespective of the substrate to which there were adhered when compared to non-polarized and M2 macrophages confirming that the M1 macrophages were activated. All macrophage phenotypes responded to C1q with a significant upregulation in phagocytic index compared to control macrophages (*p* ≤ 0.01). There was an average 3.7-fold higher phagocytic index in non-polarized macrophages and 1.7 and 1.9-fold higher phagocytic index in M1 and M2 macrophages, respectively (Fig. 1). While the fold enhancement in C1q-dependent phagocytosis was higher in non-polarized macrophages compared to polarized macrophages in this set of experiments, there is considerable experiment-to-experiment variation. These data further confirm that C1q broadly enhances phagocytic activity in a variety of macrophages.

### C1q-dependent enhancement of phagocytosis of EAIgG occurs independently of LAIR-1

**Fig. 2:**
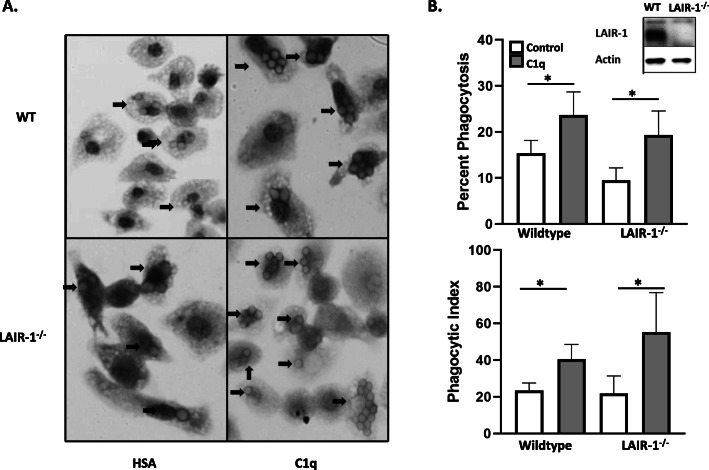
C1q-dependent enhancement of phagocytosis does not require LAIR-1. BMDM were obtained from wild-type and LAIR-1 knockout (LAIR-1^−/−^) mouse femurs. **a** Wildtype and LAIR-1^−/−^ BMDM were adhered to 4 μg/mL HSA (control) or C1q, and the phagocytosis assay was performed as described in the legend for Fig. 1. Arrows point to phagocytic cells. **b** LAIR-1 was not detected by western blot in the LAIR-1 −/− macrophages (inset). Percent phagocytosis was determined by dividing the number of macrophages ingesting at least one target by the total number of macrophages counted, multiplied by 100. The phagocytic index was calculated as the total number of red blood cells in 100 macrophages, multiplied by 100. Scoring was performed blinded with at least 200 cells per well. Bars represent the average of eight individual wells from two separate experiments + standard error of the mean. * *p* ≤ 0.05, paired student’s t-test

While the role of C1q in the enhancement of phagocytosis has been well documented, the signaling mechanisms involved in the pathway remain mostly unknown. Leukocyte-associated Ig-like receptor 1 (LAIR-1) is a collagen receptor expressed on hematopoietic cells that has been shown to interact with C1q and inhibit Toll-like receptor (TLR) activity in monocytes and dendritic cells [9–11] as well as inhibit signaling through the T-cell receptor [14]. To determine if C1q-dependent enhancement of phagocytosis of EAIgG required LAIR-1, LAIR-1^−/−^ macrophages were compared to wildtype macrophages after control and C1q stimulation. Cell morphology was similar between control and LAIR-1^−/−^ BMDM, and the cells ingested a comparable number of targets at baseline, which increased with C1q stimulation (Fig. 2a). There was a 1.5-fold increase in phagocytosis in C1q-treated wildtype macrophages when compared to control treatment (Fig. 2b). In comparison, there was a 2-fold increase in phagocytosis in C1q-treated LAIR-1^−/−^ macrophages compared to control-treated cells (Fig. 2b). Wildtype macrophages responded to C1q with a 1.7-fold increase in phagocytic index over control-stimulated wildtype macrophages. In comparison, LAIR-1^−/−^ macrophages responded to C1q with a 2.5-fold increase in phagocytic index over control cells. Deficiency in macrophage LAIR-1 was confirmed by western blot (Fig. 2b, inset). These results demonstrate that C1q-dependent enhancement of phagocytosis is independent of LAIR-1.

### Adhesion to C1q alters ERK1/2 regulation

**Fig. 3:**
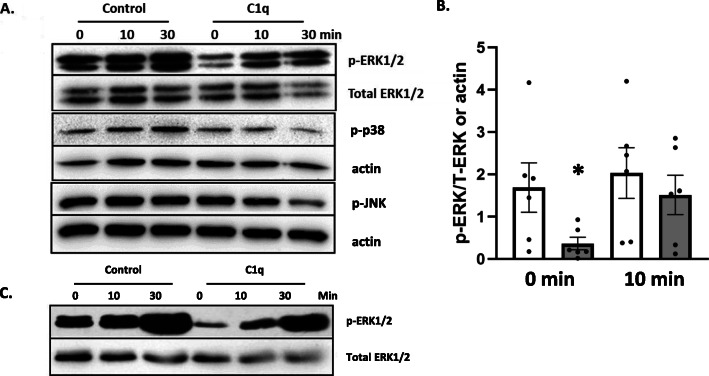
C1q regulates ERK1/2 phosphorylation in IC-stimulated BMDM and THP-1. a BMDM were adhered to 4 μg/mL HSA (control) or C1q for 30 min and then incubated with IC for an additional 0, 10 min, or 30 min. After the indicated time, cells were lysed and levels of phospho-proteins and control proteins were detected by western blot. One representative western blot from 3 to 6 replicates is shown **b** BMDM were processed as described in **a** and phosphorylated ERK1/2 (p-ERK) was normalized to either total ERK1/2 (*n* = 3) or actin (*n* = 3) loading controls at 0 and 10 min using ImageJ. Bars represent the mean of 6 individual experiments with SEM and points represent individual experiments. *p < 0.05 paired student’s t-test for time zero control versus C1q. **c** THP-1 cells were processed as described in **a** and one representative blot out of three is shown. All western blot images were cropped to display relevant bands. Corresponding uncropped full-length blots are included in supplementary Fig. 2

To further investigate cell signaling following macrophage adhesion to C1q, we utilized a phospho-kinase array to measure phosphorylation of multiple targets in BMDM adhered to control protein or C1q. To activate FcγR signaling, we further treated cells with BSA anti-BSA immune complexes (IC) for 5 min. C1q-adherent macrophages had a reduction in ERK1/2 phosphorylation compared to the control cells after 30 min adhesion to C1q as well as after 5 min stimulation with IC (Fig. S1). To validate the array, ERK1/2 phosphorylation was measured by western blot following 30 min adhesion to C1q and after addition of IC. BMDM adherent to C1q had an average 4.6-fold reduction in ERK1/2 phosphorylation compared to control BMDM (Fig. 3a and b, time zero control versus C1q). As expected, ERK1/2 was phosphorylated following treatment with IC in both control and C1q treated cells after 10 and 30 min at levels similar to control cells. There was no significant difference in ERK1/2 phosphorylation after 10 min IC stimulation between control and C1q treated cells (Fig. 3b). These data demonstrated that BMDM adherent to C1q have reduced ERK1/2 phosphorylation compared to controls, but rapidly activate ERK1/2 comparable to control cells following activation of FcγRs. Similar results were shown in the monocyte-like human cell line, THP-1 (Fig. 3c) where there was a reduction in ERK1/2 phosphorylation after 30 min of C1q stimulation when compared to the control (Fig. 3c, 0 min) and IC triggered rapid ERK1/2 phosphorylation comparable to control cells (Fig. 3c).

To determine if other MAPK phosphorylation levels were dysregulated in C1q-stimulated cells, levels of phospho-p38 and JNK were measured under conditions as described for ERK1/2 (Fig. 3a). Without any IC stimulation, there was no significant difference between control-stimulated cells and C1q-stimulated cells without IC in either phosphorylated p38 or JNK. Furthermore, the addition of IC at any time point did not reveal a significant difference in p38 or JNK phosphorylation when control-treated cells were compared to C1q-treated cells, suggesting ERK1/2-specific regulation by C1q under these experimental conditions.

### Inhibition of ERK1/2 phosphorylation prevents C1q-dependent enhancement of phagocytosis

**Fig. 4:**
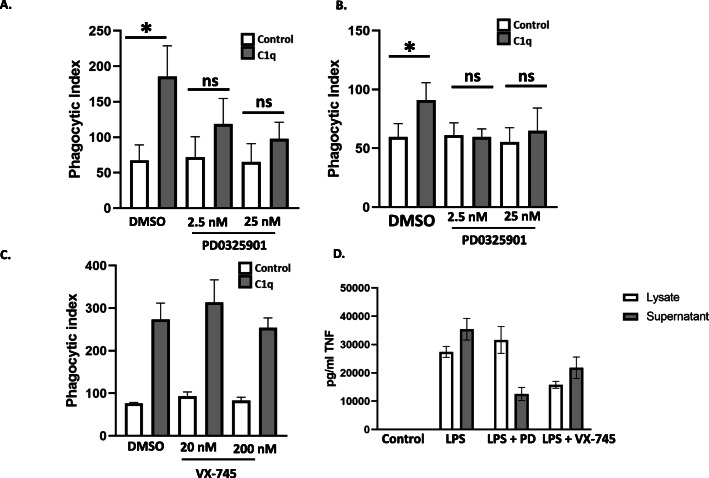
PD0325901 inhibits C1q-dependent enhancement of phagocytosis of EAIgG. a BMDM were adhered to 4 μg/mL HSA (control) or C1q for 30 min then treated with the vehicle control (DMSO) or PD0325901 for an additional 15 min. BMDM were then incubated with EAIgG and processed for phagocytosis as described in the legend to Fig. 1. Bars represent the mean of 3 individual experiments + SEM. *p ≤ 0.05 using a paired Student’s t-test. **b** THP-1 cells were processed as described in **a**. Bars represent the mean of 3 individual experiments + SEM. *p ≤ 0.05 paired student’s t-test. n.s. = not significant. **c** BMDM were adhered to control protein or C1q as described in **a** then treated with the vehicle control (DMSO) or VX-745 for 15 min. BMDM were then processed for phagocytosis. Bars represent the mean of 3 individual experiments + SEM. **d** Raw264.7 cells were either untreated or pre-treated with 250 nM PD3259901 or 200 nM VX-745 for 15 min prior to the addition of 300 ng/ml LPS for an additional 4 h. Supernatants and cell lysates were harvested and TNFα measured by ELISA. Bars represent duplicate or triplicate wells (±SD) from one experiment representative of two

Since ERK1/2 phosphorylation was differentially regulated in C1q-stimulated BMDM compared to control BMDM under conditions that result in enhanced phagocytosis, we tested the hypothesis that C1q-dependent enhancement of FcγR-dependent phagocytosis required ERK1/2 activation. We used PD0325901, a noncompetitive, specific MEK inhibitor that blocks ERK1/2 phosphorylation and activation [23]. BMDM that were adhered to C1q for 30 min and then fed EAIgG demonstrated a 2.8-fold enhancement in the phagocytic index compared to the control (Fig. 4a). Addition of the ERK1/2 inhibitor significantly reduced C1q-dependent enhancement of phagocytosis of antibody-coated particles with an average of 56.4% inhibition of the enhancement for 2.5 nM of ERK1/2 inhibitor and 74.1% inhibition of the enhancement for 25 nM of ERK1/2 inhibitor (Fig. 4a, *n* = 3) indicating that ERK1/2 phosphorylation is necessary for optimal C1q-dependent enhancement of phagocytosis in BMDM. Importantly, the ERK1/2 inhibitor did not inhibit baseline phagocytosis on the control protein (HSA), indicating that ERK1/2 is not required for FcγR-mediated phagocytosis in this system, and that this reduction in phagocytosis is specific to C1q signaling. Similar to BMDM, treatment of THP-1 with PD0325901 resulted in an inhibition of ERK1/2 phosphorylation and a complete loss of C1q-dependent enhancement of the phagocytic index when compared to control (Fig. 4b, *n* = 3). Thus, ERK1/2 phosphorylation is required for C1q-dependent enhancement of phagocytosis of antibody-coated particles in both mouse and human phagocytes. Since p38 MAPK phosphorylation was not regulated by C1q (Fig. 3), we hypothesized that inhibition of p38 would not alter C1q-dependent phagocytosis. As expected, inhibition of p38 activity using VX-745 did not inhibit C1q-dependent phagocytosis in BMDM (Fig. 4c). VX-745 was active because it inhibited LPS-dependent TNFα production that was both secreted by macrophages as well as associated with the cells (Fig. 4d). Similarly, PD0325901 inhibited LPS-dependent TNFα production from macrophages however while inhibition of secreted TNFα was detected, there was no inhibition in cell associated TNFα consistent with a reported role for ERK1/2 in regulating cleavage of TNFα from the cell surface rather than gene expression [24].

### C1q and fibronectin enhance phagocytosis through an ERK1/2-dependent mechanism

**Fig. 5:**
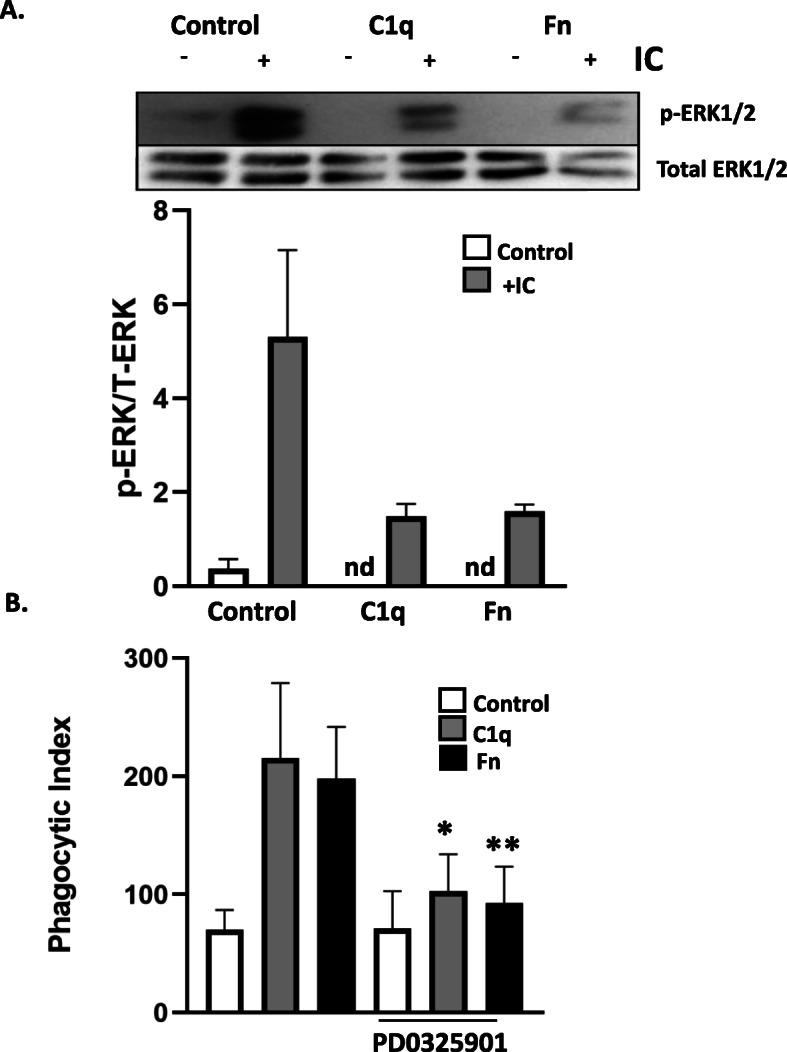
C1q and fibronectin upregulate phagocytosis of EAIgGs through a similar ERK1/2 mechanism. **a** BMDM were adhered to 4 μg/mL HSA (control), C1q, or fibronectin (Fn) for 30 min and then incubated with BSA-anti-BSA immune complexes (IC) for an additional 10 or 30 min. After the indicated time, cells were lysed and levels of ERK1/2 phosphorylation and total ERK1/2 were detected by western blot and analyzed using ImageJ. Bars represent the average of 3 individual experiments + SEM. Representative western blot depicted (top) aligns with quantification sample order (bottom). **b** BMDM were adhered to 4 μg/mL HSA (control), C1q, or Fn for 30 min and treated with the vehicle control (DMSO) or PD0325901 for 15 min, incubated with EAIgG, and processed for phagocytosis as described in previous figure legends and materials and methods. Bars represent the mean of 3 individual experiments normalized + SEM. *p ≤ 0.05 using a Student’s t-test. All western blot images were cropped to display relevant bands. Corresponding uncropped full-length blots are included in supplementary Fig. 2

Fibronectin is an extracellular matrix protein that mediates a variety of cellular functions, including the enhancement of FcγR-mediated phagocytosis [25]. Fibronectin regulates leukocyte activation largely via the family of integrin receptors, among which α5β1 and αvβ3 have been described in regulating leukocyte phagocytosis [26]. While both β1 and β2 integrins have been implicated in some C1q-dependent functions, such as mast cell activation, endothelial cell adhesion, and neutrophil superoxide production [27–29], the requirement for integrin activation in C1q-dependent phagocytosis has not been explored. To determine if C1q and fibronectin utilize similar signaling pathways for enhanced phagocytic function, we investigated the requirement of ERK1/2 phosphorylation in fibronectin-dependent phagocytosis. BMDM adherent to both C1q and fibronectin demonstrated almost no ERK1/2 phosphorylation when compared to cells adherent to the control protein, and stimulation with IC resulted in a rapid upregulation of ERK1/2 phosphorylation (Fig. 5a). BMDM adhered to C1q or Fn demonstrated a 3 and 2.8-fold increase in phagocytosis of EAIgG, respectively. Subsequent inhibition of ERK1/2 phosphorylation with PD0325901 prevented C1q- and fibronectin-dependent phagocytosis demonstrating that similar signaling pathways were utilized for enhanced phagocytosis (Fig. 5b).

### C1q-dependent ERK1/2 regulation is apparent after LPS treatment

**Fig. 6:**
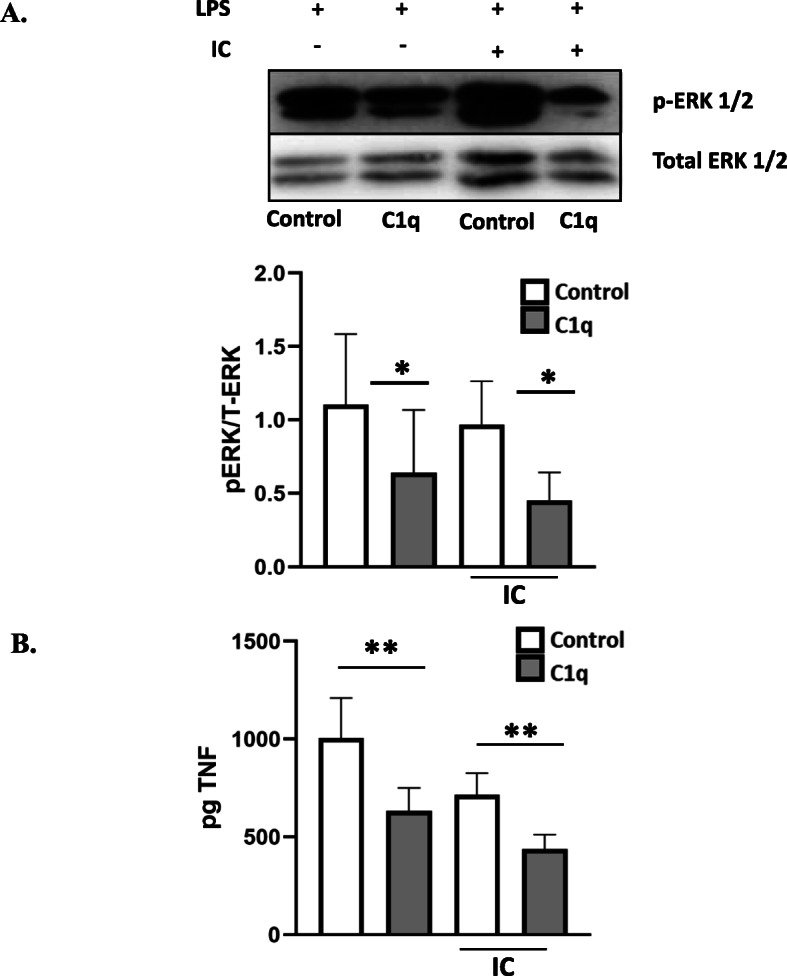
C1q downregulates ERK1/2 phosphorylation and TNFα production. BMDM were adhered to 4 μg/mL HSA (control) or C1q for 30 min, treated or not treated with IC for an additional 15 min, and then incubated with 300 ng/mL LPS for 4 h. **a** Cells were lysed and levels of ERK1/2 phosphorylation and total ERK1/2 were detected by western blot and analyzed using ImageJ. Bars represent average p-ERK divided by total ERK from 3 individual experiments + SEM. Representative western blot depicted (top) aligns with quantification sample order (bottom). *p ≤ 0.05 paired student’s t-test. **b** BMDM supernatants were collected from samples described in **a** and TNFα was measured by ELISA. Bars represent the mean of 10 individual experiments **p ≤ 0.01 paired student’s t-test. All western blot images were cropped to display relevant bands. Corresponding uncropped full-length blots are included in supplementary Fig. 2

The role of ERK1/2 in inflammation has been widely explored, and it is known that macrophage stimulation with LPS induces macrophage ERK1/2 activation involved in the progression of the inflammatory response. Furthermore, inhibition of ERK1/2 results in a significant reduction in the production of TNFα [24]. These data suggest that C1q-dependent regulation of ERK1/2 may also regulate cytokine production. To determine if C1q regulated ERK1/2 phosphorylation following activation with LPS, BMDM were adhered to C1q or control protein and stimulated for 4 h in the presence of LPS with or without IC. Adhesion to C1q led to a significant reduction in ERK1/2 phosphorylation. There was a 1.7-fold reduction in ERK1/2 phosphorylation in BMDM adherent to C1q when compared to control cells in the presence of LPS (Fig. 6a, *n* = 3). Additionally, C1q-stimulated BMDM that received IC before being stimulated with LPS demonstrated a 2.1-fold reduction in ERK1/2 phosphorylation compared to IC treated control cells. Under these conditions, C1q-stimulated cells produced significantly less TNFα. While IC-stimulated samples also demonstrate a trend in reduced TNFα production, this was not significant. C1q-stimulated cells treated with LPS in the presence and absence of IC produced 1.5-fold and 1.6-fold less TNFα compared to the control, respectively (Fig. 6b, *n* = 10).

## Discussion

Our results demonstrate that C1q and fibronectin enhance FcγR-mediated phagocytosis through a shared pathway that requires ERK1/2. Initial efforts to define the mechanism leading to C1q-dependent enhancement of phagocytosis focused on identifying the phagocytic C1q receptor however, while multiple C1q-binding proteins and/or molecules that influence C1q dependent functions have been described, not one of them has been identified as an essential receptor for enhanced phagocytic function. To add to that body of knowledge, here we demonstrated that LAIR-1, a collagen receptor required for some C1q-dependent functions, is not required for C1q-dependent phagocytosis of EAIgG. These combined results suggest there may be a redundancy in receptors utilized for C1q-dependent signaling and/or a requirement for a multimolecular signaling complex. To approach the problem in an unbiased way, we used an intracellular phospho-protein array to identify signaling intermediates downstream of the C1q receptor(s) as a means to characterize mechanism.

BMDM adhered to C1q for 30 min undergo a reduction of ERK1/2 phosphorylation compared to control cells (Fig. 3a), suggesting that either the control protein stimulates ERK1/2 phosphorylation, or a MAPK phosphatase (e.g. MKP-1) is activated during C1q stimulation. In support of the latter, cells that were adherent to heat-inactivated C1q responded similarly to cells plated on HSA; basal ERK1/2 phosphorylation was higher in cells adherent to heat inactivated C1q compared to cells adherent to non-heat treated C1q (data not shown). Subsequent immune complex stimulation led to a rapid phosphorylation (activation) of ERK1/2 in both the control cells and C1q-treated cells (Fig. 3). Further, C1q-treated cells demonstrated a greater enhancement of ERK1/2 phosphorylation since baseline ERK1/2 phosphorylation was reduced in C1q-stimulated cells (Fig. 3). While the in vitro model system used here involved cell adhesion to C1q- coated surfaces, C1q would be immobilized on a target particle in vivo. Indeed, C1q enhances phagocytosis when it is directly bound to immune complexes [30] or apoptotic cells [31]. C1q also modulates cytokine responses both when immobilized on a plate or on an apoptotic cell [31]. Furthermore, when pre-incubated with modified lipoproteins (oxidized LDL or acetylated LDL, but not native LDL), C1q enhances ingestion of atherogenic lipoproteins and modulates their subsequent metabolism [32]. Combined, these studies suggest that C1q, either immobilized on plastic or on a target particle modulate phagocyte activation in response to a variety of target particles. Interestingly, Jehle et al. [21] demonstrated enhanced ERK1,/2 phosphorylation in murine BMDM following stimulation with soluble C1q that was not associated with a target particle or immobilized on a surface. It will be important to further investigate C1q-dependent ERK1,/2 activation and inhibition when C1q is presented as a soluble protein or immobilized to a target particle.

Our data demonstrate that C1q-dependent enhancement of FcγR-mediated phagocytosis required ERK1/2, but not p38. The MAPK family regulates abundant cellular functions in innate immunity including phagocytosis and cytokine production via subfamily members ERK1/2, JNK and p38 [33]. While there are distinct roles for each MAPK in different cellular functions, there is also strong evidence of cross-talk in the signaling pathways. Our data show that C1q-dependent enhancement of FcγR-mediated phagocytosis is specific to ERK1/2 and occurs independently of p38 since inhibition of ERK1/2 significantly reduced the C1q-dependent enhancement of phagocytosis while inhibition of p38 did not. Moreover, neither JNK nor p38 phosphorylation were significantly altered with C1q stimulation. Inhibition of ERK1/2 phosphorylation via upstream inactivation of MEK1 using PD0325901 does not affect total levels of ERK protein expression, it occurs rapidly (complete inhibition within 15 min), and it persists for at least 72 h in melanoma cell lines [34]. These results remain consistent in our model where BMDM treatment with 25 nM of PD03285901 resulted in almost complete inhibition of ERK1/2 phosphorylation without affecting protein levels of ERK5(data not shown). Additionally, the use of the ERK1/2 inhibitor did not affect basal levels of phagocytosis. This is consistent with previous research by Garcia-Garcia et al. demonstrating that ERK1/2 was not required for FcγR-mediated phagocytosis [35].

Extracellular matrix molecules such as laminin and fibronectin, which bind to integrins on phagocytic cells, enhance FcγR-mediated phagocytosis similar to C1q [25, 26, 36]. Here, we demonstrated that enhanced phagocytosis via both fibronectin and C1q requires ERK1/2 since it was blocked by PD0325901 (Fig. 5). These data suggest that C1q-dependent enhanced phagocytosis may be integrin-mediated, potentially through a collagen-binding integrin. Edelson et al. demonstrated that the collagen-binding integrin α2β1 mediated C1q-dependent mast cell activation [29], and β1 integrins have also been implicated in C1q-dependent endothelial cell adhesion [28]. Using *Arpc2* −/− macrophages and the Arp2/3 complex inhibitor CK-666, Rotty et al. demonstrated that the actin nucleating ARP2/3 complex was required for macrophage integrin functions, but not for FcγR-mediated phagocytosis [37]. Since fibronectin mediated phagocytosis is regulated by integrins, and ERK1/2-dependent signaling during enhanced phagocytosis was shared between C1q and fibronectin, we investigated the dependence of integrin signaling in C1q-dependent phagocytosis using CK-666. CK-666 inhibited control FcγR-mediated phagocytosis and enhanced phagocytosis mediated by both C1q and fibronectin in our adhesion-based phagocytosis assay indicating that using methods described here, baseline phagocytosis is integrin-mediated (data not shown). Therefore, the dependence of enhanced phagocytic activity on integrins could not be assessed. C1q-mediated enhanced phagocytosis is dose dependent and plateaus at approximately 20 μg/ml immobilized C1q [4]. The data presented here demonstrate a shared ERK1/2-dependent pathway for C1q and fibronectin, and therefore we hypothesize that there would not be an additive or synergistic contribution to phagocytosis, and this should be investigated.

Finally, we hypothesized that the C1q-dependent regulation of ERK1/2 signaling may alter additional signaling pathways in addition to phagocytosis since ERK1/2 is involved in an abundance of cellular functions. We demonstrated that ERK1/2 signaling was dysregulated in cells adherent to C1q following 4-h stimulation with LPS since there was a significant downregulation in ERK1/2 phosphorylation in cells adherent to C1q. This correlated with a significant decrease in LPS-dependent TNFα production (Fig. 6). Combined, these results demonstrate that C1q stimulates ERK1/2-dependent enhanced phagocytosis of antibody-coated particles while limiting proinflammatory cytokine production, and these activities may be beneficial for enhanced clearance of immune complexes or pathogens, while limiting potentially detrimental inflammatory signaling. Continued investigation of the pathways driving C1q-dependent regulation of the immune response should contribute to development of therapeutics for autoimmune and/or chronic inflammatory diseases.

## Conclusions

The major finding from this study is the requirement for ERK1/2 in C1q-dependent phagocytosis that is independent of LAIR-1. Further, there is a conserved ERK1/2-dependent pathway for enhanced phagocytosis mediated by C1q and fibronectin. Finally, a C1q-dependent reduction in ERK1/2 phosphorylation/activation is detected after LPS stimulation that correlates with a reduction in TNFα production. The dysregulation of the immune response in the absence of C1q leads to devastating autoimmunity, and treatment options are limited in these patients. Identification of molecular mechanisms driving C1q-dependent regulation of the immune system should provide new insight into therapeutic development in autoimmunity and other chronic inflammatory disorders.

## Materials and methods

### Reagents

RPMI 1640 and Dulbecco’s Modified Eagle’s Medium (DMEM) were purchased from ThermoFisher Scientific (Waltham, MA). Penicillin/streptomycin solution was obtained from ThermoFisher Scientific and used at a final concentration of 100 U/ml penicillin G sodium, 100 μg/ml streptomycin sulfate. Fetal bovine serum (FBS) was purchased from HyClone Laboratories/GE Healthcare Life Science (Logan, UT) and then heat inactivated at 56 °C for 30 min. Bovine serum albumin (BSA) was purchased from Sigma-Aldrich (St. Louis, MO). Human C1q was purified from normal human plasma as previously described [38]. The C1q preparation was free of endotoxin as determined by the LAL Chromogenic Endotoxin Quantitation Kit purchased from ThermoFisher Scientific. Human serum albumin (HSA) was purchased from Baxter (Deerfield, IL). Ultra-pure lipopolysaccharide (LPS) was purchased from List Biological Laboratories (Campbell, CA). The ERK1/2 inhibitor PD0325901 was purchased from LC laboratories (Woburn, MA). The ARP2/3 complex inhibitor CK-0944666 (abbreviated as CK-666) was purchased from Sigma-Aldrich. The p38 inhibitor VX-745 was purchased from ThermoFisher Scientific.

### Antibodies and ELISA

The following primary antibodies were purchased from Cell Signaling (Danvers, MA): Mouse anti- p44/42 MAPK (total ERK1/2) catalog number 4696S, mouse anti-phospho-p42/44 MAPK (pERK1/2) catalog number 5726S, rabbit anti-phospho-p38 MAPK catalog number 4511, and rabbit anti-phospho-SAP/JNK catalog number 4671. HRP-conjugated secondary antibodies were purchased from Jackson ImmunoResearch (West Grove, PA). Mouse monoclonal anti-β-actin catalog number A2228 was purchased from Sigma-Aldrich. TNFα was quantified by ELISA (ThermoFisher) following manufacturer’s instructions.

### Mice

All mice were of a C57BL/6 background. Mice were housed in the Des Moines University animal care facility. Mice were used at 4–12 weeks of age as a source of BMDM. All methods were performed in accordance with protocols approved by the Des Moines University Institutional Animal Care and Use Committee. LAIR-1 deficient BMDM were generated from the femurs of LAIR-1 knockout mice generously provided by Dr. Linda Myers at the University of Tennessee Health Science Center and were harvested and passed as described below.

### Mouse cell culture

BMDM were generated from the femurs and tibias from 4 to 12-week-old C57BL/6 mice as described [39]. Briefly, the bone marrow was flushed from the bones with DMEM containing 2% heat-inactivated FBS, 10 mM HEPES, and 100 U/ml penicillin G sodium, 100 μg/ml streptomycin sulfate. Red blood cells were lysed with Ammonium-Chloride-Potassium (ACK) lysing solution (ThermoFisher Scientific). Cells were cultured in DMEM containing 10% heat-inactivated FBS, 10 mM HEPES, and 100 U/ml penicillin G sodium, 100 μg/ml streptomycin sulfate at 37 °C and 5% CO2 for 2–4 h to remove adherent cells. Non-adherent cells were collected then cultured in DMEM, 15% L929 conditioned medium, 10% heat-inactivated FBS, 100 U/ml penicillin G sodium, 100 μg/ml streptomycin sulfate, and 10 mM HEPES at 37 °C and 5% CO2. On the fourth day, the medium was replaced with fresh BMDM medium. Cells were fully mature at 7 days. Media was replenished every two to 3 days and cells were passed every 7 days (a maximum of three passes). To polarize BMDM, the culture media was first replaced with DMEM/F12, 10% heat-inactivated FBS, 100 U/ml penicillin G sodium, 100 μg/ml streptomycin sulfate, and 10 mM HEPES, and cells were incubated for 7 to 24 h. 100 U/ml IFNγ (Shenandoah Biotechnology Inc., Warwick, PA) and 100 ng/ml ultra- pure lipopolysaccharide (LPS) were added to polarize toward an M1 (inflammatory or classically activated) phenotype, and 10 ng/ml IL-4 (Shenendoah Biotechnology Inc.) was added to polarize toward an M2 (anti-inflammatory) phenotype. The cells were incubated overnight. In some experiments, polarization was confirmed by western blot for iNOS (M1) or Arginase 1 (M2), data not shown.

The THP-1 human cell line was purchased from ATCC (Manassas, VA). Cells were cultured in RPMI 1640 supplemented with 10% heat inactivated FBS, 5 μM β-mercaptoethanol (Sigma-Aldrich), and 100 U/ml penicillin G sodium, 100 μg/ml streptomycin sulfate at 37 °C and 5% CO2. Cells were split every 2–3 days to maintain the culture in log growth phase. The mouse macrophage cell line Raw264.7 was purchased from ATCC and cultured in DMEM with 10% FBS, 100 U/ml penicillin G sodium, 100 μg/ml streptomycin sulfate and 10 mM HEPES.

### Western blotting

One-well Lab-Tek chamber slides were coated for 2 h with 2 ml of 4–8 μg/ml human serum albumin (HSA) or C1q, then gently rinsed twice with sterile phosphate-buffered saline (PBS) before the addition of 10^6^ BMDM or THP-1 suspended in phagocytosis buffer (RPMI 1640 supplemented with 100 U/ml penicillin G sodium, 100 mg/ml streptomycin sulfate and 5 mM MgCl2). Samples were prepared as previously described [40]. Briefly, at the indicated time points, whole-cell lysates were collected using radioimmunoprecipitation assay buffer (RIPA buffer) supplemented with 2 μM of activated sodium vanadate, 10 mM sodium fluoride solution, 2 mM EDTA, 1 mM PMSF, a protease inhibitor tablet, and a phosphatase inhibitor tablet (Roche, Basel, Switzerland). Total protein concentration of the samples was measured by bicinchoninic assay (BCA) according to the manufacturer’s protocol (ThermoFisher Scientific) and 10–20 μg of protein were resolved using 10% SDS-PAGE under reducing conditions. Protein samples were transferred for 3 h at 300 mAmps onto a polyvinylidene difluoride (PVDF) membrane and blocked with either 5% BSA Tris-buffered saline with 0.05% Tween-20 (TBST) or 5% Milk TBST for at least 1 h. Membranes were then probed with antibodies as described in the figure legends and developed using enhanced chemiluminescence (GE Healthcare, Chicago, IL). Densitometry was performed using ImageJ software (National Institutes of Health and the Laboratory for Optical and Computational Instrumentation at the University of Wisconsin).

### Phagocytosis of EAIgG

Sheep erythrocytes were suboptimally opsonized with rabbit anti-sheep IgG (EAIgG) as previously described [41], and phagocytosis assays were performed as described [17]. Briefly, 8-well Lab-Tek chamber slides were coated with 4–8 μg/ml of HSA or C1q for 2 h. The wells were washed twice with sterile PBS before the addition of cells at a concentration of 1.25 × 10^5^–2.5 × 10^5^ cells/ml. Cells were allowed to adhere for 30 min at 37 °C and 5% CO2. ERK1/2 inhibitor PD0325901 or p38 inhibitor VX-745 was added as indicated in figure legends. EAIgGs were then added for an additional 30 min for phagocytosis to occur. After 30 min, cells were fixed with glutaraldehyde and stained with Giemsa. A blind count was performed for at least 200 cells per duplicate well. Percent phagocytosis was determined by dividing the number of macrophages ingesting at least one target by the total number of macrophages counted, multiplied by 100. The phagocytic index was calculated as the total number of red blood cells in 100 macrophages, multiplied by 100.

### Immune complex assays

To generate BSA-anti-BSA immune complexes (IC), anti-BSA antibody (Sigma-Aldrich, catalog number SAB4301142) was diluted to 0.4 mg/ml in PBS and 5 ul 10 mg/ml BSA was added to 750 ul anti- BSA and incubated at 37 °C with minimal shaking for 1 h. After the incubation, the IC were washed in sterile phagocytosis buffer. A BCA was performed on the IC to determine concentration. One-well Lab-Tek chamber slides were coated for 2 h with 2 ml of 4–8 μg/ml HSA or C1q, then gently rinsed twice with sterile phosphate buffered saline (PBS) before the addition of 10^6^ BMDM suspended in phagocytosis buffer. BMDM were adhered for 30 min on HSA or C1q before the addition of 15 μg/mL of IC for the indicated time. In some experiments 300 ng/ml LPS was added for an additional 4 h to detect cytokine production.

### Phospho-kinase Array

BMDM were stimulated with and without C1q and activated with IC for 5 min as described above for the IC assays. The phospho-kinase array was performed per manufacturers protocol (R&D Systems, Minneapolis, MN). Imaging and analysis of the blot was done using a BioRad ChemiDoc XRS imaging system.

### Statistics

Statistics were performed as described in figure legends using GraphPad Prism 8.4.3 unless otherwise indicated. Significance was determined as *p* ≤ 0.05.

## Supplementary Information

**Additional file 1: . Figure S1** Alteration in ERK1/2 phosphorylation by C1q was detected from a phospho-kinase array. **Figure S2.** Uncropped full-length blots are included for figures 3 (A and B), 5(C) and 6(D). The area that is cropped is indicated by the dashed box.

## Data Availability

The datasets used and/or analyzed during the current study are available from the corresponding author on reasonable request.

## Abbreviations

BMDM: Bone marrow derived macrophages
BSA: Bovine serum albumin
DMEM: Dulbecco’s Modified Eagle’s Medium
EAIgG: Erythrocytes suboptimally opsonized with rabbit anti-sheep IgG
ERK: Extracellular signal-regulated kinase
FBS: Fetal bovine serum
FcγR: Fcγ receptor
HSA: Human serum albumin
IC: Immune complex
IFNγ: Interferon γ
ITIM: Immunoreceptor tyrosine based inhibitory motif
JNK: Jun N-terminal kinase
LAIR-1: Leukocyte associated immunoglobulin like receptor-1
LPS: Lipopolysaccharide
MAPK: Mitogen-activated protein kinases
PVDF: Polyvinylidene difluoride
TBST: Tris-buffered saline with 0.05% Tween-20
TLR: Toll-like receptor
TNF: Tumor necrosis factor
