# Supplementary material for: Extracellular signal-regulated kinase 1/2 is required for complement component C1q and fibronectin dependent enhancement of Fcγ- receptor mediated phagocytosis in mouse and human cells
Source: BMC Immunol. 2020 Dec 14;21:61. doi: 10.1186/s12865-020-00393-6 (PMC7734837; doi:10.1186/s12865-020-00393-6)
Supplement: Supplementary file 1 — Additional file 1: . Figure S1 Alteration in ERK1/2 phosphorylation by C1q was detected from a phospho-kinase array. Figure S2. Uncropped full-length blots are included for figures 3 (A and B), 5(C) and 6(D). The area that is cropped is indicated by the dashed box. [file 12865_2020_393_MOESM1_ESM.pdf]

**Supplemental figures for:**

# Extracellular signal-regulated kinase 1/2 is required for complement component C1q and fibronectin dependent enhancement of Fcγ- receptor mediated phagocytosis in mouse and human cells

[1]Willmann, Emily A., [1]Pandurovic, Vesna, [1]Jokinen, Anna, [1]Beckley, Danielle, and [2*]Bohlson, Suzanne S. [1]Department of Microbiology and Immunology, Des Moines University, IA, USA

[2]Department of Molecular Biology and Biochemistry, University of California, Irvine, CA, USA.

# Figure Legends

**Supplemental Figure 1. Alteration in ERK1/2 phosphorylation by C1q was detected from a phospho-kinase array.** BMDM were adhered to 4µg/mL HSA (control) or C1q for 30 min, then incubated with BSA-anti-BSA immune complexes (IC) for an additional 0 min or 5 min. After the indicated time, cells were lysed and phosphorylation of 43 different proteins in duplicate were detected by the phospho-kinase array and analyzed using ImageJ. (A) Image of the phospho-kinase array with p-ERK1/2 circled in black. (B) Quantification of p-ERK1/2 from the array in (A). Bars represent the average of the duplicate samples.

**Supplementary Figure 2**. Uncropped full-length blots are included for figures 3 (A and B), 5 (C) and 6 (D). The area that is cropped is indicated by the dashed box.

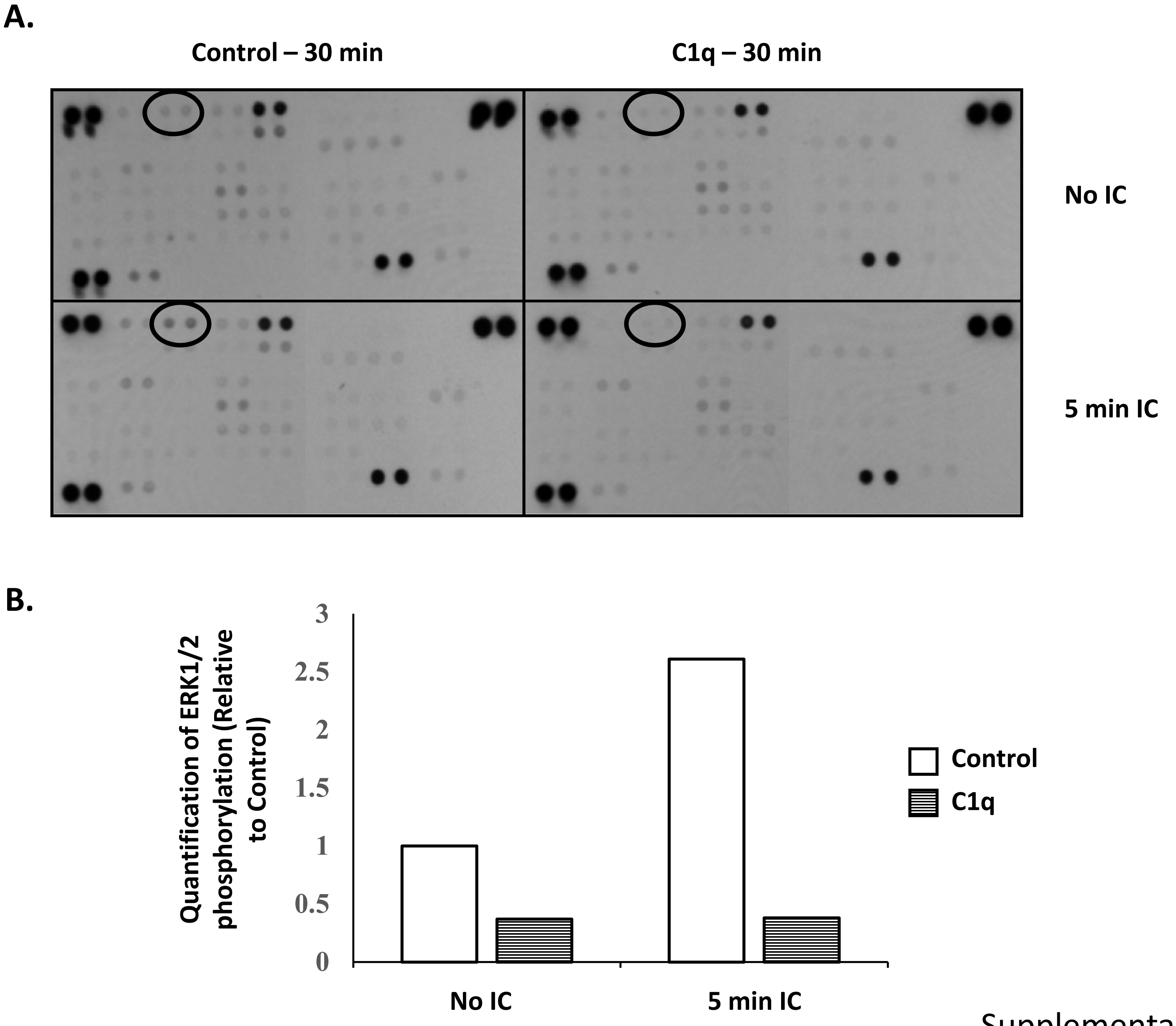


Supplemental figure 1

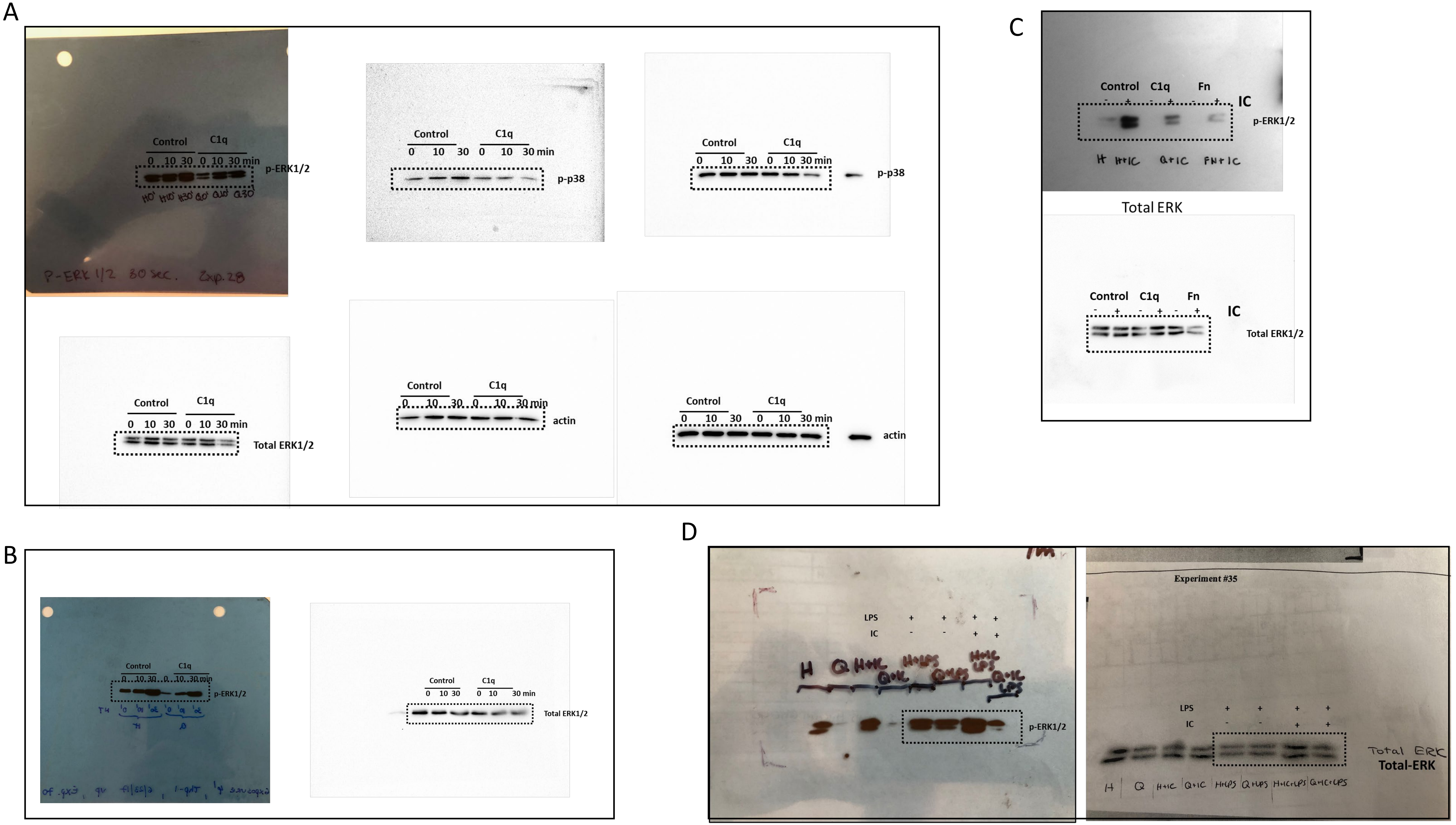
A
Control
C1q
0 10 30 0 10 30 min
p-ERK1/2
p-p38
Total ERK1/2
actin
B
Control
C1q
0 10 30 0 10 30 min
p-ERK1/2
Total ERK1/2
C
Control
C1q
Fn
IC
p-ERK1/2
Total ERK
Total ERK1/2
D
LPS
IC
p-ERK1/2
Experiment #35
Total-ERK
